# The Transformative Nature of Residential Immersive Life Skills Programs: Integrating Findings from a Five-Year Prospective Study of Program Opportunities, Youth Experiences, and Outcomes

**DOI:** 10.3390/ijerph192315865

**Published:** 2022-11-29

**Authors:** Gillian King, Amy C. McPherson, Shauna Kingsnorth, Jan Willem Gorter

**Affiliations:** 1 Bloorview Research Institute, 150 Kilgour Road, Toronto, ON M4G 1R8, Canada; 2 Department of Occupational Science and Occupational Therapy, University of Toronto, Toronto, ON M5G 1V7, Canada; 3 Dalla Lana School of Public Health, University of Toronto, Toronto, ON M5T 3M7, Canada; 4 Holland Bloorview Kids Rehabilitation Hospital, Toronto, ON M4G 1R8, Canada; 5 Department of Rehabilitation, Physical Therapy Science & Sports, University Medical Center Utrecht, Heidelberglaan 100, 3584 CX Utrecht, The Netherlands; 6 Wilhelmina Children’s Hospital, Lundlaan 6, 3584 EA Utrecht, The Netherlands; 7 CanChild, Department of Pediatrics, McMaster University, Hamilton, ON L8S 4L8, Canada

**Keywords:** active ingredients, youth with disabilities, evidence summary, participation-level intervention, personal growth, self-determination, transition

## Abstract

Youth with disabilities often experience limited opportunities to acquire the life skills needed in adulthood. As a result, life skills programs are provided to support life skill development; however, little is known about the active ingredients of these programs, and the sustainability of their effects over time. Accordingly, the aim was to synthesize the findings of a five-year study examining the opportunities, experiences, and outcomes of residential immersive life skills (RILS) programs for youth with disabilities. A multi-method prospective study was conducted involving 38 youth ages 14 to 21 with disabilities (e.g., cerebral palsy, spina bifida) attending one of three RILS programs held over three summers. Program opportunities, youth experiences, and outcomes (self-determination, self-efficacy) were assessed pre- and post-program and 3 and 12 months post-program using standardized questionnaires. Pre-program, 3-month, and 12-month follow-up interviews were held with youth and parents. This research synthesis integrates the findings from nine published articles that used a variety of qualitative, quantitative, and mixed methods approaches. RILS programs provided rich opportunities for youth to experience meaningful social connections, psychological engagement, and choice and control, which were associated with changes over time in multiple domains related to personal growth and preparation for adulthood. Overall, the findings point to the transformative power of RILS programs to propel new life directions for some youth. By creating opportunities for meaningful, challenging, and supportive experiences fulfilling basic needs for relatedness, competency, and autonomy, RILS programs motivate youth to grow and change. More study is needed of program opportunities and capacity-enhancing experiences, as well as longitudinal studies of youth life outcomes. RILS programs have appreciable value in preparing youth for the transition to adult roles and responsibilities.

## 1. Introduction

“*I would say out of the whole experience, she came back a different kid. She came back confident... Like almost like a take charge kinda personality... I didn’t think anybody could change in [X] days like that, but they can. ...Yeah, she’s definitely got more confidence, she’s got more... just direction*.” (Parent of a youth with a disability, p. 8) [1]. This quote indicates that residential immersive life skills (RILS) programs can do more than facilitate the acquisition of concrete life skills (i.e., adaptive behaviors that help people make informed decisions and manage their lives) [2]. This youth developed a sense of control, confidence, and direction she did not have previously.

Youth with disabilities are at high risk for poorer social integration in adulthood. Although they have the same life aspirations as their typically developing peers [3], they lag behind with respect to education, employment, independent living, and meaningful personal relationships [4,5]. A lack of life skills has been recognized as an important factor contributing to these poorer life outcomes [5]. Youth with disabilities experience physical and social restrictions, overprotectiveness from parents worried about their safety and emotional well-being [6], and lower expectations from both themselves and others [7]. Since youth with disabilities are widely known to experience difficulties at the time of transition to adulthood, life skills programs are offered to prepare them for life ahead.

RILS programs are a unique type of life skills program providing youth with opportunities for a range of rich learning experiences in an away-from-home group setting with peers with disabilities. RILS programs are participation-level interventions [8] that facilitate the development of life skills to support youth in the transition to adulthood. In these programs, youth with disabilities participate in a variety of formal and informal activities with small groups of peers (typically 8 to 10 youth). For some youth, this is one of their first opportunities to engage with others with disabilities.

Our research program has included three well-established RILS programs that share a common philosophy and curriculum. They are based on a common supportive, experiential, and participation-based philosophy co-created by the developers of these rehabilitation programs, who collaborated for over 10 years [9]. Key aspects of their curriculum reflect those of other life skills programs, including a combination of structured group education, one-to-one support, peer mentorship, and experiential sessions [10].

These programs are delivered in college residences for one- to three-week periods in the summer by interdisciplinary teams (e.g., occupational therapists, recreational therapists, nurses, personal attendants, and social workers). To attend these programs, youth must be between 14 and 21 years of age, have a child-onset disability, and have the cognitive capacity to set goals. As well, they must not have behavioral difficulties that may hinder their learning and the learning of others in the group. All youth enrolled in the programs were eligible to participate in the research synthesized here.

RILS programs are immersive in nature, unlike other life skills programs offered on a session-by-session basis. *Immersive* refers to extended opportunities provided for youth to have meaningful and challenging experiences with a group of peers, away-from-home in college residences. Other immersive participation-level programs have been found to provide compelling and potentially life-altering experiences for youth with disabilities, including outdoor adventure programs [11], physical activity participation interventions [12], and skills-based summer camps [13,14]. In these immersive programs, young people with disabilities have personally meaningful and challenging experiences together in overnight settings for extended periods, where they experience support from and connection with one another [13,14]. These experiences can encourage self-reliance, new self-understandings, and a sense of belonging or community [14].

Research on life skill programs has focused on short-term changes related to self-determination, self-concept, and preparedness for life [10,15,16] and studies have not examined the longer-term sustainability of skills acquired in the programs. As well, research on life skills programs has seldom examined the active ingredients of these programs, including the opportunities they provide for learning, growth, and social experiences. As a result, we began a program of research to investigate both the underlying processes and longer-term outcomes of RILS programs. The purpose of the present article is to provide a synthesis of findings from a five-year prospective cohort study.

### 1.1. Guiding Conceptual Frameworks

In a 10-year program of research, which involved pilot studies and the prospective cohort study, we examined the active ingredients and outcomes of three RILS programs in order to understand how these long-standing programs ‘work’. This research was guided by several conceptual frameworks, including a life span and complex systems perspective [17,18,19], which proposes that the health development process is complex and non-linear, and sensitive to environmental exposures and experiences.

Second, self-determination theory (SDT) was used to conceptualize the types of meaningful experiences youth could have. SDT considers needs for relatedness, competency, and autonomy to be fundamental motivators of behavior [20]. Third, we adopted an *Opportunity-Experience-Outcome* (OEO) approach, based on a Developmental Health Model of relationships between environmental opportunities and growth-enhancing experiences [21]. This conceptual model has been informed by various literatures, including research on environmental qualities, the developmental benefits of participation in activities, and quality of experience [21]. The model links environmental qualities to psychological engagement and developmental benefits, through the underlying mechanisms of opportunity, choice, and support [22]. Thus, exposure to particular types of environmental opportunities can increase the likelihood that participants will have growth-enhancing experiences that can lead, over time, to changes in their life outcomes [4]. This OEO approach guided the program components we studied and how we conceptualized, and thus examined, their linkages or associations. The OEO approach allowed us to consider opportunities, experiences, and outcomes as distinct yet interrelated constructs that can be measured both quantitatively and qualitatively. This OEO approach aligns with the context-mechanism-outcome framework used in realist evaluation [23], where experiences are the mechanisms by which program environments lead to improvements in outcomes.

Based on these guiding frameworks, we investigated the active ingredients of RILS programs, namely the OEO components: (1) what they offer (opportunities), (2) how they are ‘received’ (youth experiences), and (3) their benefits (youth outcomes). Thus, our interest was in understanding whether and how the design of RILS program environments might foster meaningful youth experiences that, over time, might contribute to optimal life outcomes for youth.

### 1.2. Preliminary Research: Grounding the Prospective Cohort Study

Prior to the large-scale prospective study reported here, we conducted several preliminary studies, including a survey of youth who had previously attended a RILS program [24]; interviews with youth [25] and service providers [26,27]; and a pilot study to trial the measurement of opportunities, experiences, and outcomes [28] and trajectories of change in youth [29].

These studies provided preliminary information about OEO associations, life outcomes for youth associated with program involvement, and the types of personal growth youth experienced. First, with respect to OEO associations, in a pilot study service providers indicated that the residential group format and opportunities for intense learning and peer social interaction were associated with life-changing experiences and youth empowerment, as well as personal changes in life skills, self-confidence, self-understandings, and self-advocacy [27]. Second, with respect to the range of possible life outcomes, a retrospective survey of 78 alumni from a RILS program indicated they had acquired and consolidated life skills as a result of program participation, were engaged in meaningful social relationships, were taking responsibility for managing their own lives, making choices, and were independent in their decision making [24]. Third, regarding the transformative power of RILS programs, a qualitative pilot study of youth and parent perceptions indicated different trajectories of changes unique to each youth [29]; these trajectories concerned significant personal growth through enhanced self-determination, self-efficacy, and self-advocacy. These initial studies provided evidence for the feasibility of the larger-scale prospective study, including the measurement of opportunities, experiences, and outcomes.

### 1.3. Research Program on RILS Interventions: The Prospective Cohort Study

Following an integrated Knowledge Translation (iKT) approach, a group of researchers and service providers from three pediatric rehabilitation organizations in Ontario Canada engaged in a funded five-year program of research [9]. Qualitative and quantitative data were collected for each of three annually run RILS programs, delivered in the summer months at three different sites, thus providing three cohorts of annual data. Participants ranged in age from 14 to 21 years and had a variety of diagnoses, including cerebral palsy, spina bifida, brain injury, and communication disorders.

Data collection involved (a) ethnographic observation of various program activity settings and researcher completion of a standardized measure of opportunities [30], (b) youth completion of a quantitative measure of their experiences in these activity settings [31], and (c) outcome measures of self-determination and self-efficacy administered pre- and post-intervention, and 3 and 12 months later. In addition, interviews were conducted pre-program and at 3 and 12 months post-program with youth and parents to examine expectations, experiences, and outcomes, and service providers were interviewed to investigate intended opportunities.

Data were analyzed quantitatively, qualitatively (e.g., thematic analysis), and using mixed methods. The aim of this research synthesis is to integrate the findings from a series of research publications, each based on a subset of the collected data from multiple sites. A conceptual integration of theory-based evidence, highlighting common findings arising from different methods and respondents, strengthens our ability to draw conclusions about the active ingredients and value of RILS programs [32], as well as their transformative aspects. Delineating the active ingredients of RILS programs can contribute to evidence-informed design and delivery of life skills interventions, particularly those with a residential or immersive format. The specific objectives of this article were to synthesize the evidence from nine research publications regarding (1) RILS program *opportunities*, (2) youth *experiences*, and (3) youth *outcomes*, and (4) integrate the findings and discuss implications for research and clinical practice.

## 2. Findings

Table 1 provides a summary overview of each of the nine research publications, its type (observation, quantitative outcome, interview), research objectives, data analysis methods, participants, nature of the OEO components examined, and quantitative assessment tools. There were five interview studies employing various types of qualitative analyses, two studies combining observations of program opportunities with interviews (analyzed using mixed methods), one solely quantitative article employing a comparison group of non-RILS youth, and one interview study also using quantitative data from both RILS and non-RILS youth (also analyzed using mixed methods). Two of the articles employed data collected observationally from on-site visits to the programs, three articles employed quantitative outcome measures completed by youth, and seven articles focused on parent, youth, and/or service provider perspectives ascertained in interviews. Thus, we leveraged various qualitative, quantitative, and mixed methods approaches to address different objectives concerning the OEO components. In the following sections, we describe the OEO components and how they were operationalized, then synthesize the findings for each component.

### 2.1. RILS Program Opportunities

*Opportunities* refer to environmental qualities of activity settings—the environmental affordances that lead to various kinds of youth experiences, such as engagement, effort, and interest [41,42]. In turn, these in-program experiences facilitate personal growth and development [43].

Important observable physical and social characteristics of activity settings, derived from the literature in psychology, disability studies, architecture, sociology, and pediatric rehabilitation, include opportunities for choice, discovery and learning, having fun, and social interaction [44]. The Measure of Environmental Qualities of Activity Settings (MEQAS-48) is a reliable and valid measure based on this literature, capturing Opportunities to Interact with Peers, and for Choice, Personal Growth, and Cooperative Group Activity, along with other types of opportunities and place-based qualities [30]. Psychometric properties of all measures are included in Appendix A.

**Figure 1:**
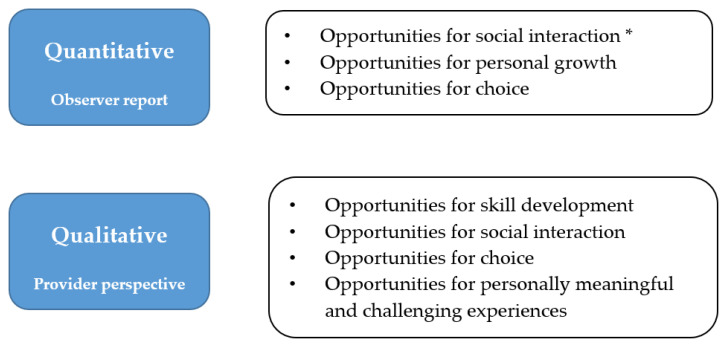
Evidence for RILS Program Opportunities. * A combination of three MEQAS scales: opportunities to interact with adults, with peers, and for cooperative group activity. This figure refers to findings from several articles [33,37].

Opportunities provided by the RILS programs were assessed using the MEQAS-48 and service providers’ perceptions (see Figure 1 and Table 1). Quantitatively, important opportunities provided by RILS programs included those for social interaction (including opportunities to interact with adults, with peers, and for cooperative group activity), personal growth, and choice. These opportunities reflect youth needs for self-determination, namely relatedness or social connection, competency, and autonomy. As shown in Figure 1, there was substantial overlap between the qualitative and quantitative findings, indicating that service providers accurately perceived the opportunities provided by the programs. In addition, service providers reported that RILS programs provided opportunities for meaningful and challenging experiences, as service delivery was individualized to provide appropriate challenges for youth. These findings indicate that RILS programs are a complex participation-based intervention, providing a program environment rich in opportunities.

### 2.2. Youth Experiences

From a context-mechanism-outcome perspective, *experiences* are the mechanisms by which program environments lead to improvements in outcomes for youth after the program has ended. Learning and development are commonly considered to result from meaningful context-specific experiences, including feelings of choice [45], challenge, self-understanding, and self-expression [46], as well as feelings of accomplishment, sense of meaning, psychological engagement, and social belonging [31]. Data used to understand youth experiences were captured both quantitatively and qualitatively.

**Figure 2:**
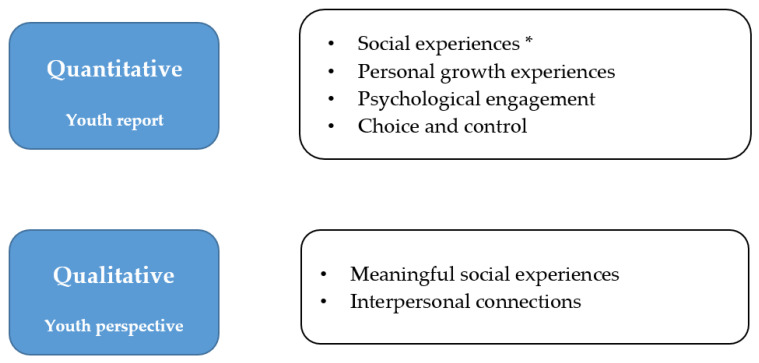
Evidence for Youth Experiences in RILS Programs. * A combination of two SEAS scales: social belonging and meaningful interactions. This figure refers to findings from several articles [34,35,37].

Quantitatively, we used the measure of Self-Reported Experiences of Activity Settings (SEAS) [31], to comprehensively capture youths’ in-the-moment participatory experiences, including their perceptions of personal growth, psychological engagement, social belonging, meaningful interactions, and choice and control. As shown in Figure 2, youth self-reports using the SEAS indicated that RILS programs provided high levels of these experiences, particularly social experiences (a combination of social belonging and meaningful interactions) and choice and control [37]. Qualitatively, youth interviews also indicated they had meaningful social experiences and formed interpersonal connections in the programs [34,35]. Thus, RILS programs provided psychologically engaging experiences and appeared to meet youth needs for relatedness (social experiences), competency (personal growth experiences), and autonomy (choice and control), as outlined by SDT [20] and the Developmental Health Model [21].

### 2.3. Youth Outcomes

Life skills programs for youth with disabilities typically examine *outcomes* related to self-determination and self-efficacy [16,47]. Accordingly, youth were asked to complete both the ARC Self-Determination Scale [48] and the General Self-Efficacy Scale [49] pre- and post-program and at 3- and 12-month follow-ups.

**Figure 3:**
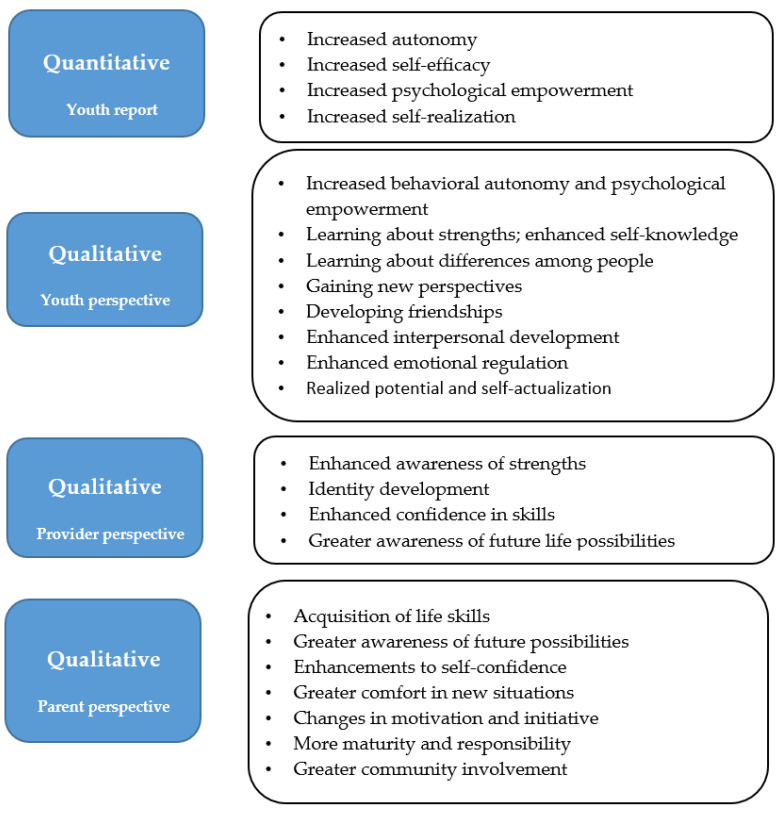
Evidence for Youth Outcomes from RILS Programs. This figure refers to findings from several articles [1,33,34,36,37,38,39,50].

We also asked youth and their parents to take part separately in interviews before and after the programs (3 and 12 months later), and interviewed service providers about intended outcomes. As shown in Figure 3, there was a wide range of youth outcomes related to learning and personal growth. Quantitatively, RILS youth reported increased autonomy, psychological empowerment, and self-realization (measured by the ARC), along with self-efficacy. Compared to a group of youth who took part in a non-residential life skills programs, RILS youth had significantly improved autonomy post-program, which was maintained at a 12-month follow-up [38,39]. As well, significant changes were found over time on self-efficacy and psychological empowerment [37].

The qualitative data indicated that youth had *new learnings or realizations* about their strengths, identity and/or sense of self, and about others. They also gained new perspectives and views of future life possibilities. This new awareness of life possibilities was reported by all stakeholders, a robust indication that RILS programs support youth transition preparedness, which is the overarching program goal. Youth-reported outcomes related to *personal growth* included developing friendships and interpersonal relationships, and realizing their potential. As well, interviews with youth held 3 and 12 months after the program indicated that they experienced changes in behavioral autonomy and in aspects of psychological empowerment at 3 months post-program, whereas at one year there was a greater emphasis on changes in self-realization [38]. Different aspects of psychological empowerment were also emphasized at the two time points, with psychological empowerment discussed 3 months post-program not only in terms of gaining confidence (as at 12 months), but also in terms of reassurance and dealing with challenges.

In addition to acquisition of life skills and enhancements to self-confidence, parents discussed a broad array of higher order outcomes for their children, including positive changes in maturity and adaptability (i.e., more comfort in new situations), increased motivation, initiative, and responsibility, along with greater community involvement [1]. Parents are more able to observe these aspects of personal growth than service providers, and have a life course perspective not yet germane to youth.

To summarize, the perspectives of the different stakeholder groups were informative, as they were aligned conceptually and yet showed differences. Youth reported changes in self-awareness, perspectives of others, social relationships, and personal growth (emotional regulation and realized potential). Service providers also indicated changes in youths’ self-awareness; in addition, they reported increased confidence in skills, and enhanced preparation for the future. Unlike youth, service providers did not comment on social relationships; however, anecdotal reports indicate service providers are aware that some youth maintain friendships formed during the program. It may be that service providers see the social benefits of RILS programs as incidental to the therapeutic effects they are working to enhance. Overall, RILS program participation was associated with changes in a range of life domains, including relationships, independent living, and community participation [1].

## 3. Discussion

Three main findings from this research synthesis are discussed here: (a) associations/linkages among opportunities, experiences, and outcomes, (b) the range of youth outcomes observed, and (c) transformative change due to RILS program participation. Each main finding was supported by our previous work, adding credence to our conclusions and supporting analytical generalization [32].

### 3.1. Associations among Opportunities, Experiences, and Outcomes (OEO)

In accordance with our OEO conceptual framework, we examined statistical associations among opportunities, experiences, and outcomes [37]. From this study [37], we concluded that it is important for RILS programs to create environments that promote youth belonging, personal growth, choice, and/or psychological engagement because these experiences were related to changes in autonomy and empowerment. Furthermore, since these experiences were associated with particular types of opportunities, we concluded that it is important and possible to intentionally design opportunities for social interaction, personal growth, choice, and psychological engagement.

Qualitative evidence for linkages among opportunities and experiences was found in a study of youths’ after-hours social experiences [34]. This study highlighted the importance of the immersive, group nature of a residential, away-from-home youth transition program, particularly the value added by the after-hours program component. The after-hours social experiences of most importance to youth were learning about strengths from working together, and having meaningful individual and group conversations. These experiences were related to various benefits: learning about differences among people, gaining new perspectives and new knowledge about oneself, and developing friendships and a sense of “family”. Thus, youth indicated the overnight aspect of RILS programs and opportunities for unstructured social interaction helped them to learn about both themselves and others, and to feel a sense of belonging and community [34]. This corresponds to previous work on the importance of the environment and social interaction to the development of positive identity and well-being [51].

### 3.2. A Range of Youth Outcomes

The varied set of outcomes that could be experienced by youth was perhaps the most unexpected finding from our research. Youth outcomes encompassed far more than the changes in self-determination and self-efficacy we had set out to examine both quantitatively and qualitatively. Youth experienced different benefits, depending on their personal needs, existing abilities, and the things they were looking for from program involvement. As well, the qualitative findings indicated that youth also changed in ways they did not anticipate or plan for, particularly changes in how they viewed themselves.

We hypothesize that changes in one area have the potential to facilitate other changes. For example, feeling more self-confident might be associated with concomitant changes in self-awareness or appreciation of others. According to Rusk’s Synergistic Change Model, lasting positive change can arise when an intervention cultivates mutually supportive interactions among various psychosocial elements (e.g., awareness, mindsets, goals, relationships) that are strong enough to create a new stable pattern of behavior [52].

### 3.3. RILS Programs as Facilitating Transformative Changes in Youth

Across our body of work, participation in RILS programs has been described as a ‘transformative experience’ and ‘turning point’. Turning points are significant life events, experiences, or realizations that lead to a fundamental shift in the meaning or direction of a person’s life [53]. Thus, participation in a RILS program can be a life-changing experience for some youth [29], leading to greater awareness of life possibilities, enhancements in self-confidence and self-efficacy, and accelerated personal growth [24,25,28]. It is important to note that youth varied in the extent of their personal growth and that some youth did not experience benefits due, in part, to lower levels of parent support for changes once they had returned to their home environment [29,50]. Parents differed in their capacity to support the development and maintenance of their youth’s personal growth [29].

RILS programs therefore have the potential to start a process of change that propels youth on a new trajectory in life. In some cases, changes that were already underway were accelerated for youth; in other instances, youth and parents stated that the observed changes would not have occurred without program participation, as participation provided new learnings, realizations, and insights. This is illustrated by the following quote from a youth asked whether they would recommend the program to others: “*Yes. I would. And I would tell them that it’s ... an experience that I think every person should have. … It’ll change the way you view things because maybe you go into that not knowing you can do certain things and you walk out of that feeling a whole lot more pride ... and independence ... and maybe it just changes the course of your life*” [38].

RILS programs can act as a springboard for youth to attain future positive life outcomes, if they continue on a trajectory of growth, which is influenced by environmental factors, events, and experiences, including parental support for changes begun in the RILS programs. “*Although RILS programs served to kick start changes for some youth, these changes were not always fully realized due to their life situations and, as a result, initial benefits disappeared for some youth. RILS program effects, therefore, are not guaranteed, nor are gains always sustainable over time or transferable to the home and community environment*” (p. 9) [1].

## 4. Strengths and Limitations of the Research

Strengths of this body of work include its theoretical and conceptual basis, prospective nature, and use of qualitative, quantitative, and mixed methods approaches. Other strengths are the wealth of data collected through observations of activity settings in three programs over three years; interviews at multiple time points over a year period with youth and parents, as well as interviews with service providers; and use of a battery of validated measurement tools to assess youth experiences and outcomes. Convergence among the findings supports the conclusions drawn in this synthesis.

Limitations include the involvement of a group of young people who have intrinsic motivation to develop their life skills, as they consented to take part in the programs. There is a risk of respondent bias with respect to their parents, as parents who consented to participate may have been more confident of their youth’s ability to do well in the program. The observational and experiential findings are limited by the sessions chosen; however, multiple observations and youth self-reports of in-session experiences were collected. The timeline of 12 months may have not been sufficient to capture emergent changes or trajectories of change for youth. Limitations for the quantitative analyses include a small sample size and lack of randomization to a control group, although a non-RILS comparison group was used in the analysis of outcome data.

## 5. Research Implications

The research implications concern conceptual frameworks relevant to intervention, study design, and measurement. Conceptually, the findings indicate the value of assessing context-mechanism-outcome linkages (operationalized as OEO) using both quantitative and qualitative approaches. This approach permits the identification of constellations of variables that can inform the design and delivery of interventions. In addition, the findings speak to the value of assessing experiences related to Self-Determination Theory and can be understood using Rusk et al.’s [52] Synergistic Change Model, where interactions among a variety of mutually reinforcing psychosocial elements are viewed as necessary to achieve lasting change. Thus, future research may likely benefit from adopting a complex systems view and exploring sets of program elements thought to be pivotal to change.

With respect to the design of studies to understand how changes arise from intervention, this work indicates the value of a longitudinal approach, as follow-up interviews at 3 and 12 months post-program indicated variability in the sustainability of program effects; in addition, new changes emerged over time as youth applied their skills, negotiated new challenges, and experienced new life events. This indicates the importance of longitudinally examining diverse outcomes emanating from program participation, reflecting a consideration of person-environment transactions over time [54] and the individual nature of the challenges and environments of youth. In this regard, a lot can be learned from studies of typically developing youth, as youth with disabilities experience similar developmental trajectories to youth without disabilities [4].

With respect to methods, the findings substantiate the value of using mixed-method approaches to capture the perspectives of multiple stakeholder groups. The qualitative findings provided rich information attesting to multifaceted benefits for youth, and the session-specific quantitative measures captured program opportunities and youth experiences in-the-moment. Furthermore, observations of opportunities in the program context, and examination of the after-hours social context, provided important perspectives on what is actually afforded to youth; these context-based aspects of the research reflect an ethnographic sensibility [55].

Ideas warranting future investigation include examining youth outcomes over follow-up periods longer than one year, placing greater emphasis on measuring program opportunities, and developing quantitative measures able to detect multifaceted changes in youth—not just changes in self-determination or self-efficacy. Measures that capture facets of a single construct do not pick up the variety of changes mentioned in the qualitative interviews: “*Measures of self-determination and self-efficacy are typically used to evaluate the outcomes of transition programs, but these measures do not capture the diversity of outcomes or even the most important aspects of preparedness*” (p. 9) [1].

## 6. Clinical Implications

This work contributes to best practices by identifying essential features of an effective participation-level intervention. Use of the findings can support evidence-informed practice and service planning, leading to improved life outcomes for transition-aged youth with disabilities. A RILS resource guide, based on the findings, is freely available for service providers https://hollandbloorview.ca/RILSguide (accessed on 19 September 2022).

RILS programs are marketed as promoting youth independence, which ignores the fundamental interdependence of all people [56]. These programs could be marketed more appropriately as supporting multifaceted personal growth by engaging youth in meaningful, challenging, and socially immersive experiences with peers with disabilities, where youth learn with and from one another. It may be useful to explain the broader benefits of program participation to youth and parents, in addition to youth being able to gain concrete life skills. By creating opportunities for capacity-enhancing experiences that fulfill basic psychological needs for relatedness, competency, and autonomy [20], RILS programs can motivate youth to grow and change.

The value of the social experiences afforded by the RILS group-based and overnight format may be underestimated by service providers, and could also be used in marketing the program to youth and parents. The findings point to the importance of immersing youth in social settings, harnessing the social aspects of a group-based program, and using an experiential and participation-based approach simulating real-world environments [57].

Last, there are implications for decision-makers and funders of life skills programs with respect to the benefits of providing real-world, immersive experiences for youth. They should be informed that RILS programs have clear value in the eyes of all relevant stakeholders (i.e., service providers, parents, and youth themselves), as they support youth to gain the outlooks, skills, and competencies needed to pursue desired adult life goals. Although RILS programs are resource intensive due to the staffing required, their duration, and overnight nature, the programs are considered to be cost-effective as they can be a springboard for life-changing experiences, community participation, and mental health and wellness outcomes for youth.

## 7. Conclusions

RILS programs are a complex, participation-based intervention with multiple active ingredients that can mobilize important developmental changes in youth with disabilities. RILS programs provide an ideal setting to prepare youth for their futures by equipping them with necessary skills, mindsets, self-awareness, and self-confidence. In our view, the challenging yet meaningful and supportive RILS program environment fosters multifaceted, synergistic types of change, which are related to youths’ basic needs for connection, competency, and autonomy. In conclusion, this research illustrates the importance of designing opportunity-filled program environments that facilitate capacity-enhancing experiences and personal growth in youth.

## Figures and Tables

**Table 1:** Overview of Studies Examining Components of RILS Programs.

Reference and Type of Study	Research Objectives	Data Analysis Methods	Participants	Components Examined			Assessment Tools ^a^
				Opportunties	Experiences	Outcomes	
King, McPherson et al. [33] Observation and interview	Observed program opportunities and service providers’ perceptions of opportunities	Mixed methods	7 service providers	✓		✓	MEQAS
King, Hartman et al. [34] Interview	Meaning of after-hours social experiences	Thematic analysis of photo elicitation-based interviews	5 youth		✓	✓	
Duff, McPherson et al. [35] Interview	The method and practice of teaching in programs	Qualitative analysis using inductive and deductive approaches	9 youth		✓		
Duff, McPherson, and King [36] Interview	Youth’s thinking of their own emotions and other’s emotions	Thematic analysis	9 youth			✓	
King, McPherson et al. [37] Observation and quantitative outcome	Opportunity-experience link and experience-outcome link	Quantitative analysis of standardized measures	29 youth	✓	✓	✓	MEQAS SEAS ARC GSE
King, Kingsnorth et al. [1] Interview	Parents’ views of the benefits of programs	Deductive thematic analysis	10 parents			✓	
King, Kingsnorth, and Tajik-Parvinchi [38] Interview and quantitative outcome	Changes in aspects of self-determination over time	Prospective mixed methods with a comparison group of non-RILS youth	27 youth			✓	ARC
Tajik-Parvinchi, Kingsnorth and King [39] Quantitative outcome	Youth changes in self-determination and self-efficacy due to program participation	Quantitative analysis with a comparison group of non-RILS youth	38 youth (27 RILS and 11 non-RILS youth)			✓	ARC GSE
McPherson, King et al. [40] Interview	Parents’ expectations and aspirations regarding program participation over time	Inductive thematic analysis	12 parents		✓		

^a^ Measure of Environmental Qualities of Activity Settings (MEQAS-48), Self-reported Experiences of Activity Settings (SEAS), ARC Self-Determination Scale (Adolescent Version), General Self-Efficacy Scale (GSE). Note: Psychometric properties of these measures are included in Appendix A.

## Appendix A. Psychometric Properties of Measures Used in the Research Program

**ARC Self-Determination Scale (Adolescent Version)**

The 72-item ARC is one of the most frequently used measures of self-determination [58], measuring four characteristics of self-determination: Autonomy, Self-realization, Psychological Empowerment, and Self-regulation [48]. The scale has demonstrated adequate reliability and validity in the measurement of self-determination for adolescents with cognitive disabilities [59]. Internal consistency is high (Cronbach’s alpha = 0.90) and criterion-related validity is adequate (r = 0.25 to 0.5) [48].

**General Self-Efficacy Scale (GSE)**

This 10-item measure has been used in numerous research studies to examine perceived self-efficacy, defined as the capacity to take action and deal with life stressors [49]. The GSE is reliable and valid, with test–retest reliabilities ranging from *r* = 0.55 to 0.75 and internal consistencies typically ranging from 0.75 to 0.91 [60]. The GSE has been used evaluatively, and has a 4-point Likert response scale, yielding a score ranging from 10 to 40; higher scores represent better self-efficacy. The GSE has been used with both adults and adolescents, including those with chronic conditions such as cerebral palsy [61,62].

**Self-reported Experiences of Activity Settings (SEAS)**

The 22-item SEAS measures key aspects of the experiences of youth with or without physical disabilities in activity settings: experiences of Personal Growth, Psychological Engagement, Social Belonging, Meaningful Interactions, and Choice and Control [31]. The SEAS has good to excellent internal consistency reliability (Cronbach’s alphas from 0.71 to 0.88) and moderate test–retest reliability (mean scale ICC = 0.68) as expected due to changes in activity settings over time. Construct validity was demonstrated by the ability of the SEAS to differentiate various types of activity settings and participation partners. The measure is available at https://research.hollandbloorview.ca/research-education/bloorview-research-institute/outcome-measures (accesssed on 19 September 2022).

**Measure of Environmental Qualities of Activity Settings (MEQAS-48)**

The MEQAS-48 [30] is an observer-rated measure of qualities and affordances of activity settings, available at https://research.hollandbloorview.ca/research-education/bloorview-research-institute/outcome-measures (accesssed on 19 September 2022). Observers rate the extent to which various qualities are present in an activity setting using a 7-point scale (7 = to a very great extent; 1 = not at all). The MEQAS-48 is an expanded version of the original 32-item measure [44], based on a more diverse sample of activity settings, including those in life skills programs. The MEQAS-48 has seven opportunity scales, including opportunities for choice, privacy/relaxation, interaction with peers, personal growth, physical activity, cooperative group activity, and interaction with adults [30]. The MEQAS-48 also contains two scales capturing the aesthetic qualities of the environment, namely comfortable place-related qualities and pleasant physical environment. An initial version of the MEQAS [44] containing 32 items (MEQAS-32) displayed very good to excellent internal consistency (Cronbach’s alphas for the scales ranging from 0.76 to 0.96); good to excellent interrater reliabilities (ICCs ranging from 0.60 to 0.93); and good to excellent test–retest reliabilities ranging from 0.70 to 0.90. The MEQAS-48 is an expansion of the MEQAS- 32, containing more items relevant to a broader range of activity settings, including those in life skills programs. The MEQAS-48 has a good fitting 9-factor model (CFI = 0.965, RMSEA = 0.049), supporting its scale structure. Concurrent validity for the MEQAS-48 has been demonstrated through significant correlations with corresponding youth experiences, and evidence of construct validity has been demonstrated through significant predicted differences for active physical, passive recreational, and skill-based activity settings [30].
